# Gene expression profiling in sinonasal adenocarcinoma

**DOI:** 10.1186/1755-8794-2-65

**Published:** 2009-11-10

**Authors:** Dominique Tripodi, Sylvia Quéméner, Karine Renaudin, Christophe Ferron, Olivier Malard, Isabelle Guisle-Marsollier, Véronique Sébille-Rivain, Christian Verger, Christian Géraut, Catherine Gratas-Rabbia-Ré

**Affiliations:** 1 Inserm, UMR 892, Nantes, F-44007, France; Université de Nantes, UFR Médecine et Techniques Médicales, Nantes, F-44000, France; 2 Service de Médecine du Travail et des Risques Professionnels, CHU de Nantes, Nantes, F-44093, France; 3 Service d'Anatomie Pathologique, CHU de Nantes, Nantes, F-44093, France; 4 Université de Nantes, UFR Médecine et Techniques Médicales, EA Biométadys, Nantes, F-44093, France; 5 Service ORL, CHU de Nantes, Nantes, F-44093, France; 6 Université de Nantes, UFR Médecine et Techniques Médicales, Plateforme Puces à ADN-OGP, Nantes, F-44000, France; 7 Université de Nantes, UFR Médecine et Techniques Médicales, Laboratoire de Biomathématiques-Biostatistiques, Nantes, F-44000, France; 8 Consultation des Pathologies Professionnelles, CH Hôtel-Dieu, Rennes, F-35000, France; 9 Service de Biochimie, CHU de Nantes, Nantes, F-44093, France

## Abstract

**Background:**

Sinonasal adenocarcinomas are uncommon tumors which develop in the ethmoid sinus after exposure to wood dust. Although the etiology of these tumors is well defined, very little is known about their molecular basis and no diagnostic tool exists for their early detection in high-risk workers.

**Methods:**

To identify genes involved in this disease, we performed gene expression profiling using cancer-dedicated microarrays, on nine matched samples of sinonasal adenocarcinomas and non-tumor sinusal tissue. Microarray results were validated by quantitative RT-PCR and immunohistochemistry on two additional sets of tumors.

**Results:**

Among the genes with significant differential expression we selected *LGALS4, ACS5, CLU, SRI and CCT5 *for further exploration. The overexpression of *LGALS4, ACS5, SRI*, *CCT5 *and the downregulation of *CLU *were confirmed by quantitative RT-PCR. Immunohistochemistry was performed for LGALS4 (Galectin 4), ACS5 (Acyl-CoA synthetase) and CLU (Clusterin) proteins: LGALS4 was highly up-regulated, particularly in the most differentiated tumors, while CLU was lost in all tumors. The expression of ACS5, was more heterogeneous and no correlation was observed with the tumor type.

**Conclusion:**

Within our microarray study in sinonasal adenocarcinoma we identified two proteins, LGALS4 and CLU, that were significantly differentially expressed in tumors compared to normal tissue. A further evaluation on a new set of tissues, including precancerous stages and low grade tumors, is necessary to evaluate the possibility of using them as diagnostic markers.

## Background

Sinonasal adenocarcinoma is a rare cancer which usually develops in the ethmoid sinuses. It mainly develops amongst 30 to 85 year old men, with a high frequency around 60. The incidence of this type of cancer was estimated by the IARC (International Agency for Research on Cancer) at 0.7/100 000 in China to 1.4/100 000 in USA and 1.5/100 000 in France, and it has been reported to account for 3% of head and neck tumors [1,2]. This cancer is recognized as an occupational cancer. In fact, it is well confirmed today that sinonasal adenocarcinoma is highly correlated with duration and level (3.5 mg/m^3^) of wood dust exposure [3,4]. As such, woodworkers have very high risks of nasal cancer (Standard Mortality Ratio: 310, 95% CI, 160-560) [5,6]. Other suspected risk factors include exposure to leather dust [7,8], metals such as chromium or nickel [9,10], and formaldehyde, although the epidemiological data regarding this chemical are partly conflicting [4,11]. In contrast to most other head and neck cancers, alcohol and tobacco do not seem to be risk factors [12]. Although the etiology of sinonasal adenocarcinoma is well-defined, its wood-related pathogenesis is not clearly understood [13]. From a morphological and histopathological point of view, these tumors are mainly intestinal-type adenocarcinomas [14,15] and demonstrate characteristic changes, such as gland formation, seen in adenocarcinomas at other anatomic sites. The most common clinical symptoms (nosebleeding, rhinitis and nasal obstruction) are not specific and this explains the delay in the diagnosis and the frequency of advanced stages. The conventional treatment includes local surgery [16] associated with radiotherapy. The survival rate at 5 years is only about 50% and it is important to point out that secondary effects are considerable due to the location of these tumors [17]. Therefore, early detection and alternative treatments are necessary. This requires, however, better knowledge of the molecular mechanisms involved in the development of these tumors. Although many reports on epidemiological studies and risk factors of sinonasal adenocarcinomas have been published, only a small number of reports have been made so far on their molecular biology. As reviewed recently by Llorente *et al *[13], several groups have proceeded with molecular studies of sinonasal adenocarcinomas. However these focused on specific genes, such as *ERBB1, CCND1, ERBB2, TP53*, *K-ras, COX-2 *or *APC*, involved either in other head and neck tumors or in colorectal cancer because of morphological similarities [13,18,19]. Two groups reported comparative genomic hybridization in ethmoid sinus adenocarcinomas and revealed hot spots of chromosomal imbalances [20-22]. Global genetic modifications (micronuclei and chromosomal aberrations) were also found in buccal epithelial cells and blood lymphocytes of wood furniture workers [23]. The conclusion of all these investigations is that ethmoid sinus adenocarcinomas have their own molecular development pathway.

Thus, to identify genes involved in this pathway, we pioneered a gene expression profiling study of 9 sinonasal adenocarcinomas versus their matched normal tissue. We found 186 genes with significant differential expression. The further evaluation of several selected genes by reverse-transcription quantitative real-time-PCR (RT-qPCR) and immunohistochemistry (IHC), on two additional validation samples, confirmed the microarray data. We have hereby opened up a new field of investigation into biomarkers of this tumor type and have identified two promising candidate genes: *LGALS4 *and *CLU*.

## Methods

### Subjects

Our study included 26 patients. A first set of 19 male patients undergoing surgery for ethmoid sinus adenocarcinomas were initially included between 2004 and 2006. Following this, a second set of 7 patients whose samples were collected from 2006 to 2007 was used to complete the immunohistochemistry study.

This project was approved by the Clinical Board of the Centre Hospitalo-Universitaire of Nantes and all included patients provided written informed consent in accordance with French regulations and the Declaration of Helsinki. All patients answered a codified questionnaire regarding occupational exposures, addictive consumption and family history. Twenty three patients out of 26 were exposed to wood dust and most of them had other occupational exposures (such as solvents and pesticides) sometimes combined with tobacco and/or alcohol. Two patients were exposed to leather dust (P7, P19), whereas only one (P10) had no occupational exposure (Table 1). Patient ages ranged from 50 to 80 years with a mean age of 69 years. To date, six patients have died as a direct result of their disease (Table 1).

**Table 1:** Summary of clinical data and use of tumor samples

*Patient*	*Age*	*Dust* *exposure*^a ^*(years)*	*Tobacco*/*alcohol*	*Other*^b^	*TNM stage* ***UICC2003***[61]	*Treatment*^d^	*Outcome*^e^	*Micro-array*	*q RT PCR*	*IHC*
1	69	W (42)	+	+	T2N0 M0	S, R	A	-	+	+
2	79	W (45)	-	-	R4bN0 M0^C^	S, R	DOD	-	-	+
3	72	W (25)	+	+	R3N0 M0	S, R	A	-	-	+
4	55	W (17)	+	+	T3N0 M0	S, R	A	-	+	+
5	62	W (3)	-	+	T4bN0 M0	S	DOD	+	+	+
6	71	W (37)	+	+	R3N0 M0	S, R	A	+	+	+
7	83	L (5)	+	+	T4aN0 M0	S, R	DOD	-	-	+
8	66	W (43)	-	-	T4bN0 M0	S	DOD	+	+	+
9	76	W (27)	+	+	R3N0 M0	S, R	A	+	+	+
10	50	-	+	-	T4aN0 M0	S, R	A	+	+	+
11	75	W (43)	-	+	T3N0 M0	S, R	A	-	+	+
12	81	W (41)	-	+	T4aN0 M0	S, R	DOD	+	+	+
13	71	W (30)	-	+	T3N0 M0	S, R	A	-	+	+
14	60	W (25)	+	+	T2N0 M0	S, R	A	+	+	+
15	73	W (6)	+	-	T2N0 M0	S, R	A	-	-	+
16	68	W (32)	+	-	T2N0 M0	S, R	A	+	+	+
17	70	W (25)	-	-	T2N0 M0	S, R	A	-	+	+
18	79	W (20)	-	-	T2N0 M0	S, R	D	-	+	+
19	77	L (12)	+	+	T4aN0 M0	S, R	A	+	+	+
20	65	W (35)	-	+	T2N0 M0	S, R	A	-	-	+
21	90	W (30)	+	-	T3N0 M0	S, R	A	-	-	+
22	54	W (42)	-	-	T2N0 M0	S, R	A	-	-	+
23	68	W (31)	+	+	T3N0 M0	S, R	A	-	-	+
24	71	W (41)	+	+	T2N0 M0	S, R	A	-	-	+
25	73	W (30)	-	+	T4aN0 M0	S, R	A	-	-	+
26	75	W (9)	-	+	T4bN0 M0	S, R	DOD	-	-	+

a: dust exposure: W = wood, L = leather

b: pesticides (xylophene), solvents (acetone, formaldehyde)

c: R = recurrent tumor

d: treatment: S = surgery, R = radiotherapy post-surgery

e: DOD = death from the disease, D = death from other causes, A = alive

### Tissue specimens

Two pieces of tissue samples were obtained from each patient undergoing surgery for ethmoidal adenocarcinoma: one from the tumor and one non-tumor sample obtained from the opposite sinus at 3 to 4 cm distance (herein referred to as "normal" tissue). All samples were immediately frozen and stored at -80°C. Remaining surgical resections of tumors and normal tissue were fixed in 10% formalin and embedded in paraffin before histological examination and diagnosis according to World Health Organization recommendations [24]. Two main types of sinonasal adenocarcinoma are recognized in the ethmoid sinus based on the histological similarity to adenocarcinoma of the intestine: Intestinal Type Adenocarcinoma (ITAC) and non-Intestinal Type Adenocarcinoma (non-ITAC). ITAC can be further divided into five categories [15,25]: the "papillary-type" (well-differentiated adenocarcinoma), the "colonic-type" (moderately-differentiated adenocarcinoma), the "solid-type" (poorly-differentiated adenocarcinoma), the "mucinous--type" and the "mixed--type" composed of a mixture of the previously defined patterns. Non-ITAC are divided into low-grade and high-grade subtypes.

### RNA extraction

On each matched normal and pathological tissue specimen from patients P1 to P19, two RNA extractions were performed from about 40 frozen sections (10 μm thick) using a Total RNA and Protein Isolation kit (Macherey-Nagel, Düren, Germany) according to the manufacturer's instructions. For each sample, the first and last sections were stained with hemalun/phloxin to confirm the histology and to evaluate the percentage of tumor tissue. 10 samples had to be eliminated for microarray analysis because of necrosis or a too low percentage of non-necrotic tumor tissue (less than 50%). Six out of these ten patients were included in the validation process by RT-qPCR as this technique is more sensitive than microarrays for identifying tumor cells within a sample. The other samples were completely excluded from the molecular analysis (Table 1).

The quantity and quality of each RNA were respectively evaluated with the NanoDrop® ND-1000 spectrophotometer (Nanodrop Technologies, Wilmington, DE) and the Agilent 2100 Bioanalyser (Agilent, Santa Clara, CA). The RNAs extracted were of good quality and the RNA integrity number (RIN) was >7.5 in all cases [26].

### RNA amplification and microarray hybridization

Cancer-dedicated microarrays were prepared in-house (ADN-OGP- Microarray Platform Nantes, France) with methods previously described in detail [27,28] using 22,175 probe sets (50-mer oligonucleotides - MWG Biotech, Roissy, France) interrogating 6,864 genes involved in different types of tumors. These microarrays therefore included triplicate probes for each gene, housekeeping genes and controls.

For microarray analysis one round of amplification was conducted on 500 ng total RNA using an Amino Allyl MessageAmp^®^II aRNA Amplification kit (Ambion, Austin, TX) according to the manufacturer's instructions, and the quantity and quality of each amplified RNA (aRNA) were again evaluated. Microarrays were carried out in duplicate for both RNA extractions of each tissue except for two patients as not enough RNA was available. The targets were prepared by labeling with Cy3-dUTP aRNA from the tumor and normal tissues. In order to reduce individual variations, the reference was prepared by mixing an equal quantity of all normal tissues [29,30] and aliquots were then labeled with Cy5-dUTP (Amersham Biosciences, Piscataway, NJ). Each Cy3-dUTP sample was mixed with an equal amount of Cy5-dUTP reference sample and the mixture was applied to microarray slides for hybridization at 40°C for 16 h [27]. The slides were then washed twice at room temperature for 2 min with 2× SSC and 0.1% SDS, for 2 min with 1× SSC, and twice for 2 min with 0.2× SSC and scanned at 10 μm/pixel resolution by ScanArray^®^ExpressHT (PerkinElmer Life Sciences, Boston, MA).

### Microarray data analysis

Scanned signals were quantified from all microarrays by GenePix Pro software version 5.1 (Axon Instruments, Union City, CA) and consolidated expression values were performed by MADSCAN software in five steps [30,31]. The information was extracted from the features close to the background or saturated and normalization was performed by the rank invariant and lowest fitness method with spatial normalization. Outlier values were eliminated with the spots in triplicate and biological replicates. To identify genes differentially expressed in tumor samples, a two-class comparison analysis by Significance Analysis of MicroArray (SAM) [32] was performed on data filtered by differences between normal and pathological tissue medians as previously described [30] and genes with differential expression were visualized using Cluster [33] and Tree view [31]. An unsupervised clustering was also performed with a hierarchical clustering algorithm [33] using the Pearson coefficient and Student test. The clusters of genes with the same regulation were functionally annotated by GoMiner [34].

The data have been incorporated into the NCBI Gene Expression Omnibus (GEO) http://www.ncbi.nlm.nih.gov/projects/geo/ and are accessible through GEO Series GPL 8957 and GSE 17433.

### cDNA synthesis and real-time PCR (RT-qPCR)

To confirm the microarray data we performed quantitative RT-PCR on selected genes using the MX4000 system and the Brilliant SYBR Green QPCR Core Reagent Kit (Stratagene, La Jolla, CA). Initially, cDNA was prepared in 20 μl using 1 μg of DNase-treated total RNA and the SuperScript III Reverse Transcriptase System (Invitrogen, Carlsbad, CA). Following a 5 fold dilution, 2 μl of each sample were used for RT-qPCR with the different pairs of primers (Additional file 1: "Primers sequences"). The following PCR cycle parameters were used: hot-start DNA polymerase activation 95°C for 10 min, 40 cycles with denaturation at 95°C for 30 sec, specific annealing temperature as indicated in "Additional file 1: Primer sequences" for 30 sec and extension at 72°C for 30 sec. Each reaction was run in duplicate. The threshold cycles, obtained from the MX4000 software, were averaged (SD<0.5). Relative expression of the target gene in the tumor versus matched normal tissue was calculated using the following equation described by Pfaffl [35], using the average Ct of three housekeeping genes: *RPLPO *(Ribosomal Protein, Large, PO), *UBC *(Ubiquitin C) and *β2M *(beta-2 microglobulin):

Relative expression per patient and per gene:

GOI = gene of interest

HK = housekeeping gene (average of Ct of the three housekeeping genes).

Eff = efficiency of the RT-qPCR obtained from the standard curve

Statistical significance was obtained using a pair-wise fixed reallocation randomization test using the REST software [36]. To insure specificity of the RT-qPCR, an agarose gel electrophoresis was initially performed to verify whether a single PCR product was generated and then a melting curve was performed at the end of each RT-qPCR. Linearity and efficiency of the RT-qPCR were checked for each gene with a standard curve of 4 logs prepared with Universal RNA (Stratagene-AGILENT, CA). Efficiency was >90% in all cases.

### Immunohistochemical analysis

Protein expression of selected genes was assessed in deparaffinized 5-μm sections of normal and pathological formalin-fixed tissue from 26 patients with sinonasal adenocarcinomas included in the study. The following antibodies were used: monoclonal antibody against human Clusterin (clone CLI-9, Alexis Corporation Lausen, Switzerland, 1:500 dilution), monoclonal antibody against human Acyl CoA synthetase 5 (ACS5) (Abnova, Jhongli City, Taiwan 1:200 dilution at 4°C overnight), polyclonal antibody against Galectin-4 (T-20) (Santa Cruz, Heidelberg, Germany, 1:50 dilution). All specimens were submitted to heat-induced antigen retrieval and processed using the EnVision Detection Kit (DAKOCYTOMATION, Trappes, France), except for LGALS4 that was processed using ABC VECTASTAIN Elite ABC Kit (Burlingame, CA), with 3,3'-diaminobenzidine as chromatogen and a hematoxylin counterstain. In each experiment, negative controls were performed by omitting the primary antibody.

## Results

### Microarray analysis

Gene expression profiles of 9 ethmoid adenocarcinomas were examined using microarrays consisting of 6864 human genes involved in many types of cancers.

**Figure 1:**
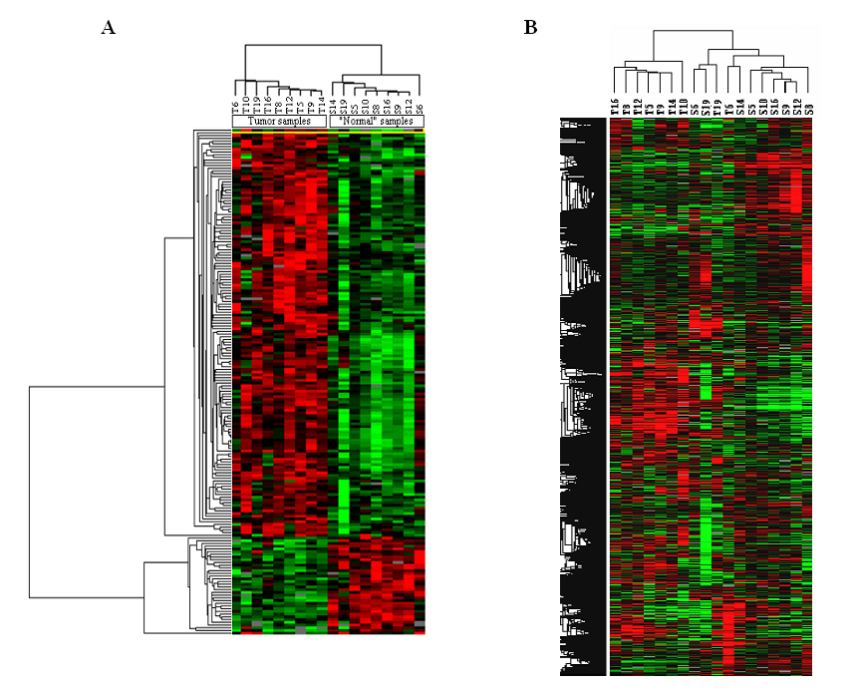
**Heat map of the two-class comparison (A) and unsupervised (B) analysis**. Expression levels are color coded with red, green, black and gray, corresponding to an increase, decrease or no change in gene expression, or missing data, respectively.

With the two-class comparison SAM, 186 genes were found to be significantly differentially expressed between ethmoid adenocarcinomas and normal sinonasal tissue. Among these 186 genes, 150 were up-regulated and 36 were down-regulated (Figure 1A and "Additional File 2: Genes with significant differential expression"). The top 59 genes (1< fold change < -1) are described in Table 2. The genes with the highest fold expression variation were selected for validation by RT-qPCR: *LGALS4 *(fold change: 3.6), *ACS5 *(fold change: 2.1), and *CLU *(fold change: -3.6). By unsupervised clustering (i.e. without any initial classification of the samples) 7 tumors out of 9 were separated from normal samples (Figure 1B). However, 5 clusters of genes with differential expression between tumor and normal samples were revealed. Using GoMiner [34] the genes involved in metabolism and biosynthesis functions were found to be overexpressed, whereas those involved in transcription, angiogenesis, cellular signaling and mitochondrial functions were down-regulated. Based on this non-supervised analysis 2 more genes with high differential expression were selected for RT-qPCR analysis: *SRI *and *CCT5*. Involved in drug resistance, these genes also featured in the list of overexpressed genes obtained from the two-class comparison analysis, with a fold change of 1.5 and 0.9 respectively.

**Table 2:** Top 59 genes differentially expressed in sinonasal adenocarcinomas after two-class comparison analysis

Accession Number	Gene Symbol	Gene annotation	Fold change (log2)
NM_006149	LGALS4	**Up-regulated genes** lectin, galactoside-binding, soluble, 4 (galectin 4)	3.084
NM_016234	ACS5	fatty-acid-Coenzyme A ligase, long-chain 5	2.621
NM_001845	COL4A1	collagen, type IV, alpha 1	1.779
NM_002228	JUN	v-jun sarcoma virus 17 oncogene homolog (avian)	1.758
NM_001816	CEACAM8	carcinoembryonic antigen-related cell adhesion molecule 8	1.732
NM_001122	ADFP	adipose differentiation-related protein	1.628
XM_067746		similar to 60 kDa heat shock protein, mitochondrialprecursor (Hsp60) (60 kDa chaperonin) (CPN60) (Heat shock protein60) (HSP-60)	1.617
NM_004591	CCL20	chemokine (C-C motif) ligand 20	1.560
BC000097	TGFBI	transforming growth factor, beta-induced, 68 kDa	1.493
NM_000393	COL5A2	collagen, type V, alpha 2	1.461
NM_003130	SRI	sorcin	1.456
NM_001153	ANXA4	annexin A4	1.434
NM_005566	LDHA	lactate dehydrogenase A	1.416
NM_005563	STMN1	stathmin 1/oncoprotein 18	1.414
NM_017958	PLEKHB2	pleckstrin homology domain containing, family B (evectins) member 2	1.409
XM_092196		similar to Cytochrome c, somatic (LOC164837), mRNA.	1.387
AF112214	MRPL13	mitochondrial ribosomal protein L13	1.370
AJ250915	HSPD1	heat shock 60 kDa protein 1 (chaperonin)	1.346
BC003623	YWHAZ	tyrosine 3-monooxygenase/tryptophan 5-monooxygenase activation protein, zeta polypeptide	1.342
NM_006111	ACAA2	acetyl-Coenzyme A acyltransferase 2 (mitochondrial 3-oxoacyl-Coenzyme A thiolase)	1.335
NM_021821	MRPS35	mitochondrial ribosomal protein S35	1.329
NM_002592	PCNA	proliferating cell nuclear antigen	1.319
NM_001827	CKS2	CDC28 protein kinase regulatory subunit 2	1.278
AB062125	TPM3	tropomyosin 3	1.224
NM_016245	DHRS8	dehydrogenase/reductase (SDR family) member 8	1.206
NM_001226	CASP6	caspase 6, apoptosis-related cysteine protease	1.194
NM_004670	PAPSS2	3'-phosphoadenosine 5'-phosphosulfate synthase 2	1.172
XM_088293		similar to cytochrome c (LOC157317), mRNA.	1.164
NM_001428	ENO1	enolase 1, (alpha)	1.145
XM_060849		similar to cytochrome C, expressed in somatic tissues(LOC128146), mRNA.	1.133
AF135381	CKLF	chemokine-like factor	1.133
X84907	ENO1	enolase 1, (alpha)	1.121
NM_005720	ARPC1B	actin related protein 2/3 complex, subunit 1B, 41 kDa	1.114
NM_021130	PPIA	peptidylprolyl isomerase A (cyclophilin A)	1.110
NM_001288	CLIC1	chloride intracellular channel 1	1.095
BC015130	CYCS	cytochrome c, somatic	1.081
NM_012255	XRN2	5'-3' exoribonuclease 2	1.068
M34664	HSPD1	heat shock 60 kDa protein 1 (chaperonin)	1.066
AF054185	PSMA7	proteasome (prosome, macropain) subunit, alpha type, 7	1.044
NM_006601	TEBP	unactive progesterone receptor, 23 kD	1.037
AF136630	CBX3	chromobox homolog 3 (HP1 gamma homolog, Drosophila)	1.023
AF274941	CKS1B	CDC28 protein kinase regulatory subunit 1B	1.013
AF320053	MYCN	v-myc myelocytomatosis viral related oncogene, neuroblastoma derived (avian)	1.006
		**Down-regulated genes**	
NM_001831	CLU	clusterin (complement lysis inhibitor, SP-40,40, sulfated glycoprotein 2, testosterone-repressed prostate message 2, apolipoprotein J)	-3.606
NM_005978	S100A2	S100 calcium binding protein A2	-2.152
S68290	AKR1C1	aldo-keto reductase family 1, member C1 (dihydrodiol dehydrogenase 1; 20-alpha (3-alpha)-hydroxysteroid dehydrogenase)	-2.102
NM_003713	PPAP2B	phosphatidic acid phosphatase type 2B	-1.966
AB000889	PPAP2B	phosphatidic acid phosphatase type 2B	-1.936
NM_001321	CSRP2	cysteine and glycine-rich protein 2	-1.916
NM_006485/	FBLN1	fibulin 1	-1.799
AF007162	CRYAB	crystallin, alpha B	-1.590
NM_002825	PTN	pleiotrophin (heparin binding growth factor 8, neurite growth-promoting factor 1)	-1.405
NM_001063	TF	transferrin	-1.256
NM_004186	SEMA3F	sema domain, immunoglobulin domain (Ig), short basic domain, secreted, (semaphorin) 3F	-1.246
NM_000424	KRT5	keratin 5 (epidermolysis bullosa simplex, Dowling-Meara/Kobner/Weber-Cockayne types)	-1.207
AF059617	PLK2	polo-like kinase 2 (Drosophila)	-1.186
NM_005596	NFIB	nuclear factor I/B	-1.149
NM_006206	PDGFRA	platelet-derived growth factor receptor, alpha polypeptide	-1.090
NM_005900	MADH1	MAD, mothers against decapentaplegic homolog 1 (Drosophila)	-1.047

### Relative expression level of selected genes

To validate the differential gene expression obtained by microarray analysis, quantitative PCR analysis of the selected genes was performed in matched sets of tumors and normal tissues. The patients used for microarray analysis and 6 additional patients were included. As RNA from normal tissue was no longer available, we used the Ct average (SD<1Ct) of all normal tissues for P8 and P19 patients to calculate the relative expression level of each gene [35].

**Figure 2:**
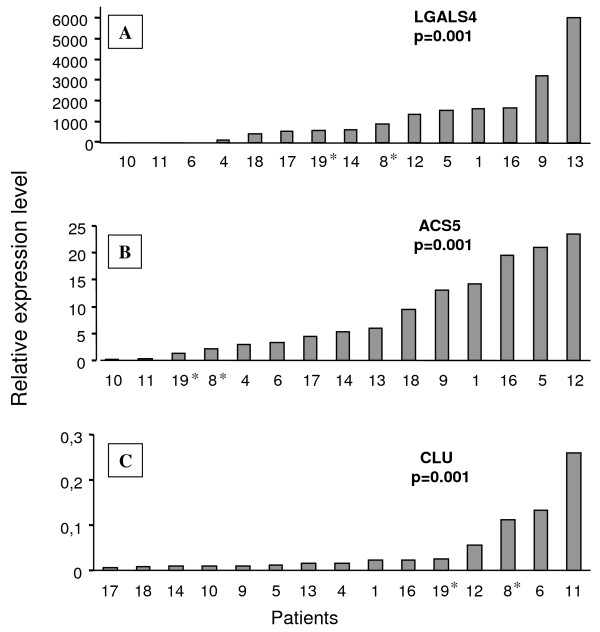
**Relative expression levels of *LGALS4*, *ACS5*, *CLU*, in tumors versus matched normal sinusal tissue as determined by RT-qPCR**. Fold change was calculated according to the equation described in the Materials and Methods with normalization against the average of three housekeeping genes, *RPLPO*, *β2 microglobulin*, and *ubiquitin C*. *tumor tissue versus average of all normal sinusal tissues (cf. RT-qPCR Results for details).

A significant differential expression in tumor tissue versus normal tissue was confirmed for all selected genes. The genes with the highest overexpression were *LGALS4 *with a mean ratio of 1309 (0.17-5993, p = 0.001), then *ACS5 *with a mean ratio of 9.48 (0.14-23.55, p = 0.001). P10 and P11 patients overexpressed neither *LGALS*4 nor *ACS5*. (Figure 2A-B). *CLU *was highly down-regulated in most of the tumors (mean ratio:0.044, 0.005-0.26, p = 0.001) (Figure 2C). Many isoforms of *CLU *have been described in the literature [37], and we quantified by RT-qPCR the main ones, i.e. the nuclear form (n-clu) and the cytosolic form (s-clu). Both were found to be down-regulated (data not shown). Regarding *SRI and CCT5*, their significant up regulation was confirmed (p = 0.0016 and p = 0.006 respectively) although the fold change was much lower ("Additional file 3: Relative expression of SRI and CCT5").

### Immunohistochemical analysis of LGALS4, ACS5 and CLU

**Figure 3:**
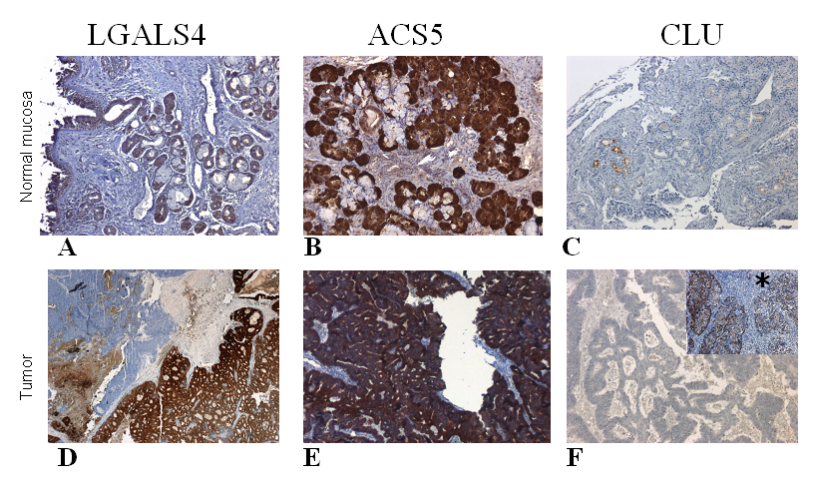
**Representative cases of LGALS4, CLU and ACS5 expression in matched normal mucosa (×100), and tumor tissue (×25)**. A-B-C: Normal sinusal mucosa immunostaining. (A-C): Weak and focal cytoplasmic staining of serous cells in a few seromucinous glands with LGALS4 and CLU. (A):Weak staining of respiratory epithelium with LGALS4. (B): Strong and diffuse immunostaining of serous cells with ACS5. D-E-F: Tumor immunostaining. (D): Poorly-differentiated "solid-type" component showing no immunoreactivity for LGALS4 while the "colonic-type" component is positive in a mixed ITAC (patient 5). (E): Example of ACS5 expression in a "colonic-type" ITAC. (F): No immunoreactivity for CLU in tumor samples (×100) except in one non-ITAC (Insert * (×25), Patient 11).

To confirm the variation in expression of the selected genes at the protein level, we performed immunohistochemical analysis of matched normal sinonasal and tumor tissues from the 15 patients used for the molecular analysis as well as from an independent set of 11 other patients, using specific antibodies for LGALS4, ACS5 and CLU (Table 3). In the normal sinusal mucosa, these three markers were expressed by serous cells of the seromucinous glands present in the lamina propria. A weak and focal cytoplasmic staining of a small number of seromucinous glands was observed with the antibodies against LGALS4 and CLU while the staining was more intense and diffuse for ACS5 (Figure 3A-B-C). Among the 26 tumors analyzed, only 2 were high-grade non-ITAC and the others were ITAC: 5 "papillary-type" (well-differentiated adenocarcinoma), 2 "colonic-type" (moderately-differentiated adenocarcinoma), 9 "mucinous-type" adenocarcinoma and 8 mixed-type adenocarcinoma (Table 3).

**Table 3:** LGALS4, ACS5 and CLU expression in 26 sinonasal adenocarcinomas (IHC analysis).

Patient	**Tumor subtypes (Barnes'classification **[25])	CLU^a^	ACS5	LGALS4
1	ITAC mixed (papillary and mucinous)	-	-	+++
2	ITAC mucinous	-	-	+++
3	ITAC mucinous	-	-	+++
4	ITAC colonic	-	+	+++
5	ITAC mixed (solid and colonic)	-	++	-/++
6	ITAC mucinous	-	+	+++
7*	ITAC mucinous	-	-	+++
8	ITAC mucinous	-	-	+++
9	ITAC mixed (papillary and mucinous)	-	+++	+++
10**	non ITAC high-grade	-	-	-
11	non ITAC high-grade	+++	++	-
12	ITAC papillary	-	+++	+++
13	ITAC mucinous	-	-	+++
14	ITAC papillary	-	-	+++
15	ITAC mucinous	-	-	+++
16	ITAC mixed (papillary and mucinous)	-	++	+++
17	ITAC papillary	-	++	+++
18	ITAC mixed (papillary, colonic and mucinous)	-	+	+
19*	ITAC mucinous	-	+	+++
20	ITAC mixed (colonic and mucinous)	-	+	+++
21	ITAC mixed (papillary and mucinous)	-	-	+++
22	ITAC papillary	-	-	+++
23	ITAC mixed (papillary and colonic)	-	-	+++
24	ITAC colonic	-	+++	++
25	ITAC papillary	-	+	+++
26	ITAC mucinous	-	-	+++

* Patients exposed to leather dust

** No occupational exposure

a- Intensity of immunostaining in tumoral cells:

+++: positivity of 75 to 100% of cells with an strong staining.

++: positivity of 25 to 75% of cells with heterogenous weak to strong staining.

+: focal and weak positivity of 1 to 25% of cells.

-: no staining.

With the LGALS4 antibody the ITAC tumor cells displayed a strong cytoplasmic and membranous staining with an additional nuclear staining in the well-differentiated adenocarcinomas. Interestingly, in a mixed ITAC sample (P5) the poorly differentiated "solid-type" component showed no immunoreactivity for LGALS4 while the "colonic-type" component was positive (Table 3 and Figure 3D). Non-ITAC samples displayed no LGALS4 expression.

For ACS5, fifty percent of the tumor samples were negative while the remaining 50% showed a weak to strong cytoplasmic staining without any correlation with the histological type or with the differentiation of the tumor (Table 3 and Figure 3E).

In contrast to normal mucosa, CLU was found to be absent in tumors except in one high-grade non-ITAC tumor (Patient P11) where there was a diffuse cytoplasmic staining (Table 3 and Figure 3F).

## Discussion

Ethmoid carcinomas are uncommon tumors recognized as an occupational disease amongst woodworkers. Current treatment with surgery and radiotherapy is unsatisfactory given the 50% survival at 5 years and the serious side effects. To better understand the molecular events involved in this tumor and to identify potentially novel markers we pioneered a gene expression profiling study of 9 sinonasal adenocarcinomas.

This study, using dedicated-microarrays containing 6864 genes previously known to be involved in cancer, allowed us to select 5 genes (*LGALS4, ACS5, CLU, SRI and CCT5*) with significant differential expression between tumors and normal tissue. We confirmed by RT-qPCR the overexpression of *LGALS4, ACS5, SRI, CCT5 *and the down-regulation of *CLU*. By IHC on an independent set of patients, we focused our interest on the genes with the highest differential expression i.e. *LGALS4, ACS5 *and *CLU*, and confirmed the results at the protein level for LGALS4 and CLU.

*The LGALS4 *gene codes for the Galectin 4 protein [38]. Galectins constitute a family of proteins containing carbohydrate recognition domains (CRD) with high affinity for β galactosides. Their complete physiological functions are not known but they have been reported to be involved in inflammation, apoptosis, cell adhesion and cell growth. LGALS4 in particular has been detected in normal epithelial cells of the oral esophagus, and in the intestinal mucosa [39,40]. In tumors, *LGALS4 *expression increases in liver, gastric, breast cancer and mucinous epithelial ovarian cancer whereas it is down-regulated in colon adenocarcinoma [41-43]. The presence of two binding sites for c-Rel, a subunit of NFκ-B, and the experimental data obtained with transgenic mice for c-Rel, suggest that LGALS4 could be a downstream component of the NFκ-B pathway, known to be involved in the regulation of tumorogenesis [44,45]. In cancer cell lines LGALS4 is expressed in highly differentiated cell lines which form polarized monolayers while undifferentiated cell lines do not express LGALS4 but Galectin1 [38,42]. In our series of ethmoid adenocarcinoma, the *LGALS4 *is the gene with the highest differential expression and our IHC data are in accordance with the literature, given that we found that LGALS4 is overexpressed in all ethmoid tumors except the high-grade non ITAC tumors which are poorly differentiated. LGALS4 expression seems to be correlated to both histological type and the differentiation status of the adenocarcinoma. This trend was confirmed by the P5 case where LGALS4 was overexpressed only in the "colonic-type" component and not in the poorly differentiated "solid-type" component of the tumor. For patient 6 (P6) we observed a strong overexpression of LGALS4 by IHC, which contrasts with the relative expression obtained by RT-qPCR (fold change 0.45). We therefore hypothesize that, in this "mucinous-type" ITAC containing numerous mucin lakes, the RNA extracted from the tissue was not representative of the tumor.

The highly conserved gene *CLU (*apolipoproteinJ, sulfated glycoprotein 2), codes for Clusterin, a sulfated glycoprotein with chaperone activity found in numerous tissues and body fluids. CLU has been reported as being involved in many biological functions such as DNA repair, cell cycle regulation and apoptosis [37,46]. CLU is described as being overexpressed in several types of cancers including colon, breast and lung cancer [37], yet a down-regulation has been found in esophageal squamous cell carcinoma, in some pancreatic, prostate or colon cancers and in HPV-negative squamous cell carcinoma of the head and neck [37,46,47], suggesting a pro-survival or a pro-apoptotic function. The recent description of several isoforms, including the nuclear form (n-CLU) and the cytoplasmic or secreted form (s-CLU), might help to resolve these apparent contradictions and to define the cellular functions of Clusterin as well as its potential use as a biomarker [48-50].

In our series of ethmoid tumors, CLU was highly down-regulated at the RNA level. Although the level of Clusterin detected by IHC in normal tissue was rather low, we confirmed the down-regulation of the protein except in one case (P11). This patient was also the one whose tumor sample showed the least down-regulation of CLU by RT-qPCR. This case is of interest because the patient was exposed to wood and, in contrast with most of the cases reported in the literature, he presented a non-ITAC tumor. The absence of Clusterin in ethmoid tumors suggests a pro-apoptotic function in normal ethmoidal tissue, possibly in response to DNA damage caused by wood dust, or other occupational exposures. It is useful to note that CLU is localized on chromosome 8p21-p12 [51]. In fact, by comparative genomic hybridization, Ariza *et al*. found losses on 8p21 in about 50% of patients with sinonasal adenocarcinomas [20]. This feature was confirmed by the study of Korinth *et al*. who reported a loss of 8p in 61% of cases [21] in a series of 42 patients. We do not know the cytogenetics of our tumors but it would be worthwhile ascertaining whether the down-regulation of CLU in the tumors studied here is due to deletion on chromosome 8p or if other mechanisms such as epigenetic regulation occur on the *CLU *gene.

*ACS5*, Acyl coenzyme A synthetase 5 (*FACL5*, E.C. 6.2.1.3.), is one isoform of the ACSs, key proteins in lipid metabolism via the activation of fatty acids in acylCoA thioesters. These esters are the metabolites for oxidation, elongation and desaturation of fatty acids as well as for the synthesis of complex lipids. ACS5 is essential for lipid metabolism but it might also play a role in intermediate metabolism and regulation of gene expression [52]. This gene has been well characterized in the small intestine mucosa by Gassler *et al *[53,54]. ACS5 is expressed in the enterocytes from the villus tip but not in the crypts and it could be involved in the differentiation and maintenance of crypt-villus axis, by inducing TRAIL apoptosis in apical villi of the mucosa. Within the context of tumorogenesis, few reports have been published on ACS5. In adenoma and adenocarcinoma of the small intestine ACS5 expression is decreased [54] while it is up-regulated in gliomas [55], in well-differentiated endometrioid adenocarcinomas [56] and in certain colorectal adenocarcinomas [57]. The RT-qPCR data in our panel of tumors revealed an increase in the expression of ACS5 (p = 0.001), eventhough it has not been confirmed by IHC. Whereas some tumors expressed strong ACS5, others had completely lost the expression of this molecule. Moreover, we could not find any correlation between ACS5 expression and histological type, differentiation or collateral exposures.

The other selected genes were not evaluated by immunohistochemistry as their variation in expression was much lower and our primary goal was to find new markers for a better characterization of these tumors with a clear etiology. Nevertheless, we confirmed the transcriptional profiling obtained with the microarray by RT-qPCR.

*SRI *(*Sorcin) *and *CCT5 (*chaperonin-containing complexe peptide 1) are less known genes. Both code for multi-drug resistance proteins and might be involved in the cell detoxification [58,59]. These genes were slightly overexpressed in our panel of tumors. This trend could be related to the chemical or particle exposures of the patients. In fact, *SRI *has also been identified by Differential Display analysis as being overexpressed in oral cancer mediated by tobacco-chewing [60].

## Conclusion

In conclusion, our transcriptomic study has enabled us to identify genes involved in sinonasal adenocarcinomas. The validation of microarray data by RT-qPCR and immunohistochemistry confirmed the significant alterations of *LGALS4 *and *CLU *expression. Because of the low incidence of these tumors we had a limited number of patients and only one without wood exposure, preventing any correlation between survival and wood exposure. Nevertheless, after validation using tissue microarrays in a large set of tumors, including pre-cancerous lesions and early stages, LGALS4 and CLU could be included in a panel of non invasive diagnostic/prognostic tests for the follow-up of woodworkers, to allow an earlier detection of lesions using a sinonasal smear.

## Competing interests

The authors declare that they have no competing interests.

## Authors' contributions

TD conceived the design of the study, performed the questionnaire, the follow up of the patients and participated in the drafting of the paper. SQ participated in the tissue collection, performed the molecular and data analyses, and contributed to the drafting of the paper. KR performed the pathological diagnoses and the immunohistochemistry interpretation. CF, OM and CV participated in the tissue collection, IGM to the microarray study. VSR and CG contributed to the design of the study and the epidemiological questionnaire. CGRR participated in the study design, supervised the project and prepared the manuscript. All authors read and approved the final manuscript.

## Pre-publication history

The pre-publication history for this paper can be accessed here:

http://www.biomedcentral.com/1755-8794/2/65/prepub

## Supplementary Material

Additional file 1 **Primer sequences**. Click here for file

Additional file 2 **Genes with significant differential expression in sinonasal adenocarcinomas, identified by two-class comparison**. Click here for file

Additional file 3 **Relative expression levels of *SRI and CCT5 *in tumors versus matched normal sinonasal tissue as determined by RT-qPCR**. Fold change was calculated according to the equation described in the Materials and Methods with normalization against the average of three housekeeping genes, *RPLPO*, *β2 microglobulin*, and *ubiquitin C*. *tumor tissue versus average of all normal sinonasal tissues (cf. RT-qPCR Results for detail). Click here for file
