# Supplementary material for: Gene expression profiling in sinonasal adenocarcinoma
Source: BMC Med Genomics. 2009 Nov 10;2:65. doi: 10.1186/1755-8794-2-65 (PMC2780459; doi:10.1186/1755-8794-2-65)
Supplement: Additional file 1 — Primer sequences. [file 1755-8794-2-65-S1.doc]

Additional file 1- Primers sequences

| Gene | Forward primer | Reverse primer | T°Annealing |
| --- | --- | --- | --- |
| CLU | tccaggacaggttcttcacc | gagagaagggcatcaagctg | 63 |
| LGALS4 | tctttgatctgtccattcgct | gatttccaatgtgtccaccc | 63 |
| ACS5 | gtgctgatagggaatgtagag | gatgggttatcatggctcct | 55 |
| BAX | aaaacacagtccaaggca | accgtgaccatctttgtg | 60 |
| PDGFRα | acttgctattacaaccacactc | tcctccacgatgactaaataatcc | 55 |
| CCT5 | ATTGCTGTGAATGCCGTCCT | GAAATCCTTGTCCACAATCACGCC | 63 |
| SRI | GCTCAGCAAGGTGTTGTGAA | GAAAAGAGGACAAGCAAAGG | 63 |
| MONDO-A | ACGATGACATGCTGTATTGG | GCTGGTGTGAAGAAAGTGTG | 60 |
| NSAP-1 | CTAGTAAAGGACCAGATGAGGC | CATTAGACGAAGATCCCATATAGG | 60 |
| β2microglobuline* | GGCATCTTCAAACCTCCATGATG | TTCACCCCCACTGAAAAAGATGA | 68 |
| RPLPO* | GATGACCAGCCCAAAGGAGA | GTGATGTGCAGCTGATCAAGACT | 68 |
| UBC* | GATTTGGGTCGCGGTTCTT | TGCCTTGACATTCTCGATGGT | 68 |

* Housekeeping gene
