# Supplementary material for: Gene expression profiling in sinonasal adenocarcinoma
Source: BMC Med Genomics. 2009 Nov 10;2:65. doi: 10.1186/1755-8794-2-65 (PMC2780459; doi:10.1186/1755-8794-2-65)
Supplement: Additional file 2 — Genes with significant differential expression in sinonasal adenocarcinomas, identified by two-class comparison. [file 1755-8794-2-65-S2.doc]

Additional file 2 - Genes with significant differential expression in sinonasal adenocarcinomas, identified by two-class comparison.

| **Gene Nb** | **Symbol** | **Annotations** | **Fold change**  **(log 2)** |
| --- | --- | --- | --- |
| NM_006149 | LGALS4 | lectin, galactoside-binding, soluble, 4 (galectin 4) | 3.084 |
| NM_016234 | ACSL5 | fatty-acid-Coenzyme A ligase, long-chain 5 | 2.621 |
| NM_001845 | COL4A1 | collagen, type IV, alpha 1 | 1.779 |
| NM_002228 | JUN | v-jun sarcoma virus 17 oncogene homolog (avian) | 1.758 |
| NM_001816 | CEACAM8 | carcinoembryonic antigen-related cell adhesion molecule 8 | 1.732 |
| NM_001122 | ADFP | adipose differentiation-related protein | 1.628 |
| XM_067746 |  | similar to 60 kDa heat shock protein, mitochondrialprecursor (Hsp60) (60 kDa chaperonin) (CPN60) (Heat shock protein60) (HSP-60) | 1.617 |
| NM_004591 | CCL20 | chemokine (C-C motif) ligand 20 | 1.560 |
| BC000097 | TGFBI | transforming growth factor, beta-induced, 68kDa | 1.493 |
| NM_000393 | COL5A2 | collagen, type V, alpha 2 | 1.461 |
| NM_003130 | SRI | sorcin | 1.456 |
| NM_001153 | ANXA4 | annexin A4 | 1.434 |
| NM_005566 | LDHA | lactate dehydrogenase A | 1.416 |
| NM_005563 | STMN1 | stathmin 1/oncoprotein 18 | 1.414 |
| NM_017958 | PLEKHB2 | pleckstrin homology domain containing, family B (evectins) member 2 | 1.409 |
| XM_092196 |  | similar to Cytochrome c, somatic (LOC164837), mRNA. | 1.387 |
| AF112214 | MRPL13 | mitochondrial ribosomal protein L13 | 1.370 |
| AJ250915 | HSPD1 | heat shock 60kDa protein 1 (chaperonin) | 1.346 |
| BC003623 | YWHAZ | tyrosine 3-monooxygenase/tryptophan 5-monooxygenase activation protein, zeta polypeptide | 1.342 |
| NM_006111 | ACAA2 | acetyl-Coenzyme A acyltransferase 2 (mitochondrial 3-oxoacyl-Coenzyme A thiolase) | 1.335 |
| NM_021821 | MRPS35 | mitochondrial ribosomal protein S35 | 1.329 |
| NM_002592 | PCNA | proliferating cell nuclear antigen | 1.319 |
| NM_001827 | CKS2 | CDC28 protein kinase regulatory subunit 2 | 1.278 |
| AB062125 | TPM3 | tropomyosin 3 | 1.224 |
| NM_016245 | DHRS8 | dehydrogenase/reductase (SDR family) member 8 | 1.206 |
| NM_001226 | CASP6 | caspase 6, apoptosis-related cysteine protease | 1.194 |
| NM_004670 | PAPSS2 | 3'-phosphoadenosine 5'-phosphosulfate synthase 2 | 1.172 |
| XM_088293 |  | similar to cytochrome c (LOC157317), mRNA. | 1.164 |
| NM_001428 | ENO1 | enolase 1, (alpha) | 1.145 |
| XM_060849 |  | similar to cytochrome C, expressed in somatic tissues(LOC128146), mRNA. | 1.133 |
| AF135381 | CKLF | chemokine-like factor | 1.133 |
| X84907 | ENO1 | enolase 1, (alpha) | 1.121 |
| NM_005720 | ARPC1B | actin related protein 2/3 complex, subunit 1B, 41kDa | 1.114 |
| NM_021130 | PPIA | peptidylprolyl isomerase A (cyclophilin A) | 1.110 |
| AF129756/NM_001288 | CLIC1 | chloride intracellular channel 1 | 1.095 |
| BC015130 | CYCS | cytochrome c, somatic | 1.081 |
| NM_012255 | XRN2 | 5'-3' exoribonuclease 2 | 1.068 |
| M34664 | HSPD1 | heat shock 60kDa protein 1 (chaperonin) | 1.066 |
| AF054185 | PSMA7 | proteasome (prosome, macropain) subunit, alpha type, 7 | 1.044 |
| NM_006601 | TEBP | unactive progesterone receptor, 23 kD | 1.037 |
| AF136630 | CBX3 | chromobox homolog 3 (HP1 gamma homolog, Drosophila) | 1.023 |
| AF274941 | CKS1B | CDC28 protein kinase regulatory subunit 1B | 1.013 |
| AF320053 | MYCN | v-myc myelocytomatosis viral related oncogene, neuroblastoma derived (avian) | 1.006 |
| NM_012073 | CCT5 | chaperonin containing TCP1, subunit 5 (epsilon) | 0.999 |
| NM_002211 | ITGB1 | integrin, beta 1 (fibronectin receptor, beta polypeptide, antigen CD29 includes MDF2, MSK12) | 0.998 |
| NM_004846 | EIF4EL3 | eukaryotic translation initiation factor 4E-like 3 | 0.983 |
| NM_006016 | CD164 | CD164 antigen, sialomucin | 0.979 |
| NM_002046 | GAPD | glyceraldehyde-3-phosphate dehydrogenase | 0.973 |
| NM_004209 | SYNGR3 | synaptogyrin 3 | 0.965 |
| NM_000581 | GPX1 | glutathione peroxidase 1 | 0.962 |
| NM_005606 | LGMN | legumain | 0.959 |
| AB049940 | MRPS5 | mitochondrial ribosomal protein S5 | 0.948 |
| AF372979 | ATP10A | ATPase, Class V, type 10A | 0.945 |
| BC017452 | RFC4 | replication factor C (activator 1) 4, 37kDa | 0.938 |
| NM_005507 | CFL1 | cofilin 1 (non-muscle) | 0.931 |
| BC029377 | DKFZp586G0123 | calcium-binding transporter | 0.930 |
| NM_000365 | TPI1 | triosephosphate isomerase 1 | 0.923 |
| XM_059584 |  | similar to evidence:NAS~hypothetical protein~putative(LOC132299), mRNA. | 0.923 |
| BC006543 | CCT5 | chaperonin containing TCP1, subunit 5 (epsilon) | 0.907 |
| NM_152255 | PSMA7 | proteasome (prosome, macropain) subunit, alpha type, 7 | 0.899 |
| NM_003404 | YWHAB | tyrosine 3-monooxygenase/tryptophan 5-monooxygenase activation protein, beta polypeptide | 0.898 |
| AB062294 | UBL1 | ubiquitin-like 1 (sentrin) | 0.889 |
| AK027801 | BCDO2 | beta-carotene dioxygenase 2 | 0.885 |
| NM_001762 | CCT6A | chaperonin containing TCP1, subunit 6A (zeta 1) | 0.884 |
| NM_000291 | PGK1 | phosphoglycerate kinase 1 | 0.877 |
| NM_002293 | LAMC1 | laminin, gamma 1 (formerly LAMB2) | 0.877 |
| NM_138635 | H2AV | histone H2A.F/Z variant | 0.871 |
| BC000431 |  | Similar to tubulin alpha 1, clone MGC:8360IMAGE:2819847, mRNA, complete cds. | 0.870 |
| NM_015878 | OAZIN | ornithine decarboxylase antizyme inhibitor | 0.859 |
| NM_005805 | PSMD14 | proteasome (prosome, macropain) 26S subunit, non-ATPase, 14 | 0.854 |
| NM_004428 | EFNA1 | ephrin-A1 | 0.845 |
| XM_067176 | LOC131055 | similar to peptidyl-Pro cis trans isomerase | 0.845 |
| BC010273 | PAICS | phosphoribosylaminoimidazole carboxylase, phosphoribosylaminoimidazole succinocarboxamide synthetase | 0.840 |
| AF161421 | LEREPO4 | likely ortholog of mouse immediate early response, erythropoietin 4 | 0.840 |
| BC021995 | SRP9 | signal recognition particle 9kDa | 0.836 |
| NM_003091 | SNRPB | small nuclear ribonucleoprotein polypeptides B and B1 | 0.833 |
| NM_004964 | HDAC1 | histone deacetylase 1 | 0.833 |
| NM_005915 | MCM6 | MCM6 minichromosome maintenance deficient 6 (MIS5 homolog, S. pombe) (S. cerevisiae) | 0.832 |
| NM_002790 | PSMA5 | proteasome (prosome, macropain) subunit, alpha type, 5 | 0.829 |
| AF077199 | LYPLA1 | lysophospholipase I | 0.828 |
| BC009524 | PSMD14 | proteasome (prosome, macropain) 26S subunit, non-ATPase, 14 | 0.827 |
| AF355127 | TUBB8-pending | tubulin, beta 8 | 0.819 |
| AF297747 | GMPS | guanine monphosphate synthetase | 0.818 |
| NM_006303 | JTV1 | JTV1 gene | 0.812 |
| NM_014423 | AF5Q31 | ALL1 fused gene from 5q31 | 0.808 |
| NM_006367 | CAP1 | CAP, adenylate cyclase-associated protein 1 (yeast) | 0.803 |
| NM_001067 | TOP2A | topoisomerase (DNA) II alpha 170kDa | 0.792 |
| NM_005274 | GNG5 | guanine nucleotide binding protein (G protein), gamma 5 | 0.790 |
| BC030981 | HMGB1 | high-mobility group box 1 | 0.781 |
| BC009218 | ENO1 | enolase 1, (alpha) | 0.777 |
| J05016 | ERP70 | protein disulfide isomerase related protein (calcium-binding protein, intestinal-related) | 0.770 |
| NM_005274 | GNG5 | guanine nucleotide binding protein (G protein), gamma 5 | 0.768 |
| NM_006713 | PC4 | activated RNA polymerase II transcription cofactor 4 | 0.766 |
| AF054183 | RAN | RAN, member RAS oncogene family | 0.749 |
| NM_015932 | C13orf12 | chromosome 13 open reading frame 12 | 0.746 |
| AF060511 |  | clone 016b10 My016 protein mRNA, complete cds. | 0.732 |
| NM_012145 | DTYMK | deoxythymidylate kinase (thymidylate kinase) | 0.732 |
| NM_002046 | GAPD | glyceraldehyde-3-phosphate dehydrogenase | 0.731 |
| NM_002786 | PSMA1 | proteasome (prosome, macropain) subunit, alpha type, 1 | 0.730 |
| D00760 | PSMA2 | proteasome (prosome, macropain) subunit, alpha type, 2 | 0.728 |
| NM_005648 | TCEB1 | transcription elongation factor B (SIII), polypeptide 1 (15kDa, elongin C) | 0.728 |
| NM_004725 | BUB3 | BUB3 budding uninhibited by benzimidazoles 3 homolog (yeast) | 0.717 |
| NM_000053 | ATP7B | ATPase, Cu++ transporting, beta polypeptide (Wilson disease) | 0.707 |
| NM_015449 | NICE-3 | NICE-3 protein | 0.705 |
| BC003375 | MRPL3 | mitochondrial ribosomal protein L3 | 0.704 |
| XM_167194 |  | similar to ARP2/3 complex 21 kDa subunit (P21-ARC)(Actin-related protein 2/3 complex subunit 3) (LOC222755), mRNA. | 0.690 |
| NM_004324 | BAX | BCL2-associated X protein | 0.676 |
| AF182290 | LSM4 | LSM4 homolog, U6 small nuclear RNA associated (S. cerevisiae) | 0.672 |
| NM_003350 | UBE2V2 | ubiquitinconjugating enzyme E2 variant 2 | 0.670 |
| XM_170597 | LOC256374 | similar to peptidylprolyl isomerase A | 0.663 |
| NM_003821 | RIPK2 | receptor-interacting serine-threonine kinase 2 | 0.662 |
| U91327 |  | chromosome 12p15 BAC clone CIT987SK-99D8 complete sequence. | 0.661 |
| NM_013232 | PDCD6 | programmed cell death 6 | 0.655 |
| AD001528 | SMS | spermine synthase | 0.648 |
| NM_006325 | RAN | RAN, member RAS oncogene family | 0.634 |
| NM_000175 | GPI | glucose phosphate isomerase | 0.631 |
| NM_002534 | OAS1 | 2',5'-oligoadenylate synthetase 1, 40/46kDa | 0.630 |
| BC007104 | PPIA | peptidylprolyl isomerase A (cyclophilin A) | 0.630 |
| AB084917 | COAS2 | cyclophilin-LC | 0.628 |
| BC003094 | TBCD | tubulin-specific chaperone d | 0.626 |
| NM_004047 | ATP6V0B | ATPase, H+ transporting, lysosomal 21kDa, V0 subunit c'' | 0.626 |
| NM_016489 | NT5C3 | 5'-nucleotidase, cytosolic III | 0.618 |
| NM_006854 | KDELR2 | KDEL (Lys-Asp-Glu-Leu) endoplasmic reticulum protein retention receptor 2 | 0.618 |
| D64154 | ADRM1 | adhesion regulating molecule 1 | 0.614 |
| NM_018480 | HT007 | uncharacterized hypothalamus protein HT007 | 0.613 |
| NM_000454 | SOD1 | superoxide dismutase 1, soluble (amyotrophic lateral sclerosis 1 (adult)) | 0.611 |
| BC003047 | YWHAH | tyrosine 3-monooxygenase/tryptophan 5-monooxygenase activation protein, eta polypeptide | 0.609 |
| XM_062890 | LOC121981 | similar to peptidyl-Pro cis trans isomerase | 0.609 |
| BC001741 | GLO1 | glyoxalase I | 0.592 |
| AF148645 | RHEB | Ras homolog enriched in brain | 0.590 |
| NM_080916 | DGUOK | deoxyguanosine kinase | 0.588 |
| NM_016139 | C7orf17 | chromosome 7 open reading frame 17 | 0.580 |
| NM_003400 | XPO1 | exportin 1 (CRM1 homolog, yeast) | 0.576 |
| NM_005216 | DDOST | dolichyl-diphosphooligosaccharide-protein glycosyltransferase | 0.575 |
| NM_001103 | ACTN2 | actinin, alpha 2 | 0.570 |
| NM_002356 | MARCKS | myristoylated alanine-rich protein kinase C substrate | 0.570 |
| NM_007217 | PDCD10 | programmed cell death 10 | 0.560 |
| NM_002415 | MIF | macrophage migration inhibitory factor (glycosylation-inhibiting factor) | 0.555 |
| BC000748 | TUBB4 | tubulin, beta, 4 | 0.551 |
| NM_002726 | PREP | prolyl endopeptidase | 0.493 |
| AL031668/NM_007367/NM_016732 | RALY | RNA binding protein (autoantigenic, hnRNP-associated with lethal yellow) | 0.474 |
| NM_005796 | NUTF2 | nuclear transport factor 2 | 0.470 |
| NM_006082 | K-ALPHA-1 | tubulin, alpha, ubiquitous | 0.455 |
| NM_006863 | LILRA1 | leukocyte immunoglobulin-like receptor, subfamily A (with TM domain), member 1 | 0.448 |
| NM_005770 | SERF2 | small EDRK-rich factor 2 | 0.446 |
| U31520 | MAN2A1 | mannosidase, alpha, class 2A, member 1 | 0.445 |
| NM_006602 | TCFL5 | transcription factor-like 5 (basic helix-loop-helix) | 0.441 |
| NM_006936 | SMT3H1 | SMT3 suppressor of mif two 3 homolog 1 (yeast) | 0.416 |
| NM_002607 | PDGFA | platelet-derived growth factor alpha polypeptide | 0.412 |
| NM_017812 | FLJ20420 | hypothetical protein FLJ20420 | 0.397 |
| NM_025197 | CDK5RAP3 | CDK5 regulatory subunit associated protein 3 | -0.409 |
| AK128868 | AHNAK | AHNAK nucleoprotein (desmoyokin) | -0.424 |
| NM_004740 | TIAF1 | TGFB1-induced anti-apoptotic factor 1 | -0.441 |
| NM_018849 / NM_018850 | ABCB4 | ATP-binding cassette, sub-family B (MDR/TAP), member 4 | -0.456 |
| NM_006139 | CD28 | CD28 antigen (Tp44) | -0.499 |
| NM_000929 | PLA2G5 | phospholipase A2, group V | -0.514 |
| BC032800 | FXYD1 | FXYD domain containing ion transport regulator 1 (phospholemman) | -0.527 |
| NM_006764 | IFRD2 | interferon-related developmental regulator 2 | -0.542 |
| NM_004672/NM_145319 | MAP3K6 | mitogen-activated protein kinase kinase kinase 6 | -0.551 |
| NM_000213 | ITGB4 | integrin, beta 4 | -0.561 |
| D38552 | KIAA0073 | KIAA0073 protein | -0.568 |
| NM_032870 | C6orf111 | chromosome 6 open reading frame 111 | -0.578 |
| AK001842 | FLJ10980 | hypothetical protein FLJ10980 | -0.653 |
| NM_006258 | PRKG1 | protein kinase, cGMP-dependent, type I | -0.671 |
| NM_018281 | FLJ10948 | hypothetical protein FLJ10948 | -0.704 |
| AJ277460 | CLSTN3 | calsyntenin 3 | -0.738 |
| L36531 | ITGA8 | integrin, alpha 8 | -0.751 |
| NM_000611 | CD59 | CD59 antigen p18-20 (antigen identified by monoclonal antibodies 16.3A5, EJ16, EJ30, EL32 and G344) | -0.819 |
| NM_003798 | CTNNAL1 | catenin (cadherin-associated protein), alpha-like 1 | -0.913 |
| NM_006765 | TUSC3 | tumor suppressor candidate 3 | -0.919 |
| NM_005900 | MADH1 | MAD, mothers against decapentaplegic homolog 1 (Drosophila) | -1.047 |
| NM_006206 | PDGFRA | platelet-derived growth factor receptor, alpha polypeptide | -1.090 |
| NM_005596 | NFIB | nuclear factor I/B | -1.149 |
| AF059617 | PLK2 | polo-like kinase 2 (Drosophila) | -1.186 |
| NM_000424 | KRT5 | keratin 5 (epidermolysis bullosa simplex, Dowling-Meara/Kobner/Weber-Cockayne types) | -1.207 |
| NM_004186 | SEMA3F | sema domain, immunoglobulin domain (Ig), short basic domain, secreted, (semaphorin) 3F | -1.246 |
| NM_001063 | TF | transferrin | -1.256 |
| NM_002825 | PTN | pleiotrophin (heparin binding growth factor 8, neurite growth-promoting factor 1) | -1.405 |
| AF007162 | CRYAB | crystallin, alpha B | -1.590 |
| NM_006485 / NM_006486 / NM_001996 | FBLN1 | fibulin 1 | -1.799 |
| NM_001321 | CSRP2 | cysteine and glycine-rich protein 2 | -1.916 |
| AB000889 | PPAP2B | phosphatidic acid phosphatase type 2B | -1.936 |
| NM_003713 | PPAP2B | phosphatidic acid phosphatase type 2B | -1.966 |
| S68290 | AKR1C1 | aldo-keto reductase family 1, member C1 (dihydrodiol dehydrogenase 1; 20-alpha (3-alpha)-hydroxysteroid dehydrogenase) | -2.102 |
| NM_005978 | S100A2 | S100 calcium binding protein A2 | -2.152 |
| NM_001831 | CLU | clusterin (complement lysis inhibitor, SP-40,40, sulfated glycoprotein 2, testosterone-repressed prostate message 2, apolipoprotein J) | -3.606 |
