# Supplementary figures and images for: Gene expression profiling in sinonasal adenocarcinoma
Source: BMC Med Genomics. 2009 Nov 10;2:65. doi: 10.1186/1755-8794-2-65 (PMC2780459; doi:10.1186/1755-8794-2-65)

## Slide 1
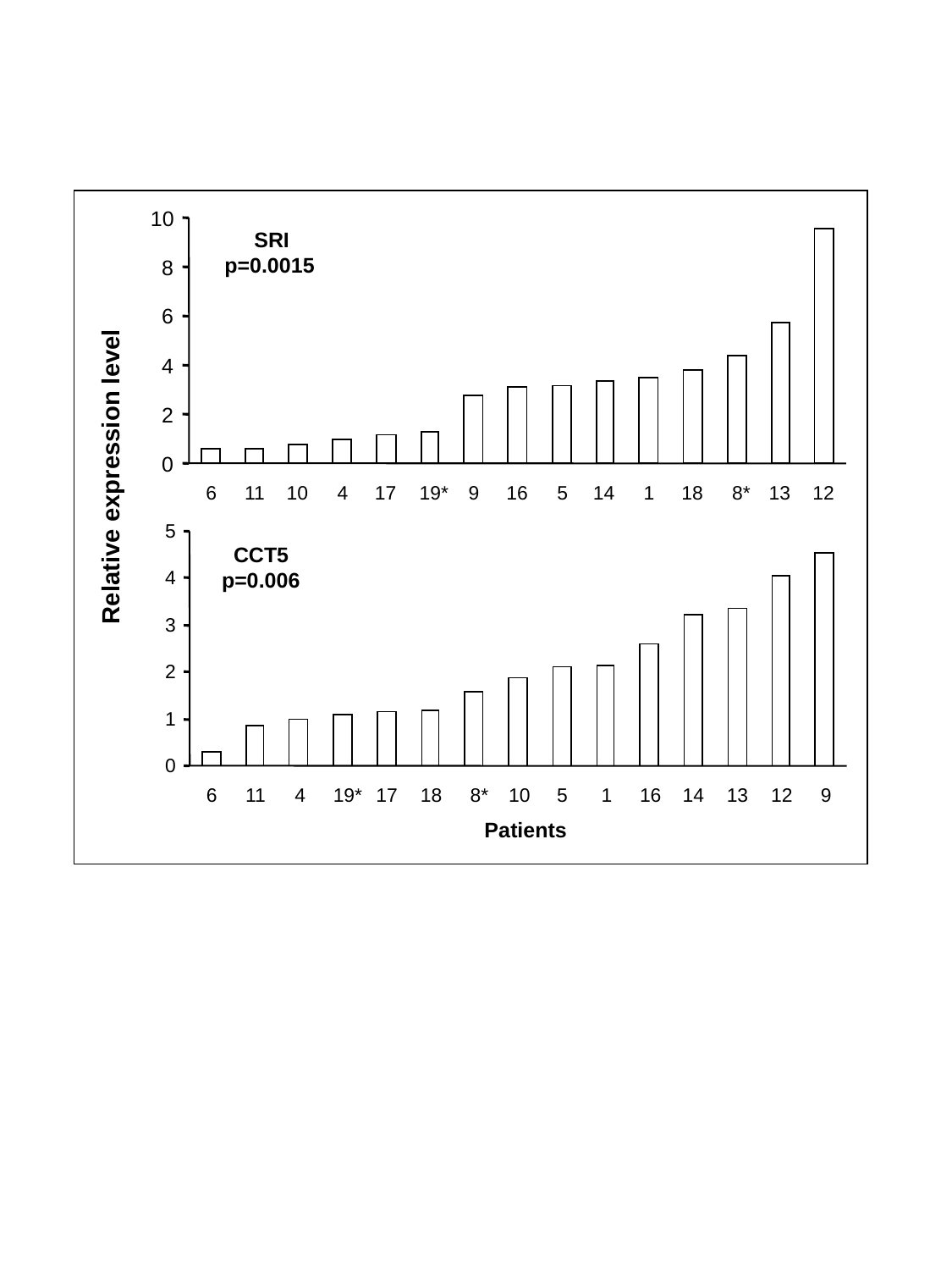

10
 SRI
p=0.0015
8
6
4
2
0
Relative expression level
6
11
10
4
17
19*
9
16
5
14
1
18
8*
13
12
5
 CCT5
p=0.006
4
3
2
1
0
6
11
4
19*
17
18
8*
10
5
1
16
14
13
12
9
Patients

Supplement: Additional file 3 — Relative expression levels of SRI and CCT5 in tumors versus matched normal sinonasal tissue as determined by RT-qPCR. Fold change was calculated according to the equation described in the Materials and Methods with normalization against the average of three housekeeping genes, RPLPO, β2 microglobulin, and ubiquitin C. *tumor tissue versus average of all normal sinonasal tissues (cf. RT-qPCR Results for detail). [file 1755-8794-2-65-S3.ppt]
